# Antitubular Basement Membrane Antibody Disease Associated with Nivolumab Infusion and Concomitant Acute Pyelonephritis Leading to Acute Kidney Injury : a Case Report and Literature Review

**DOI:** 10.1155/2023/6681756

**Published:** 2023-04-03

**Authors:** Ahmad Oussama Rifai, Kristin M. Denig, Tiffany Caza, Shana M. Webb, Sarah Rifai, Sarah Khan, Sally Dahan, Samaa Alamin

**Affiliations:** ^1^The Virtual Nephrologist INC, Lynn Haven, FL, USA; ^2^Hypertension Kidney and Dialysis Specialists, Panama City, FL, USA; ^3^Arkana Laboratories, Little Rock, AR, USA; ^4^Alabama College of Osteopathic Medicine, Dothan, AL, USA; ^5^University of Medical Sciences and Technology, Sudan (UMST), P.O. Box 12810, Khartoum, Sudan

## Abstract

Antitubular basement membrane (anti-TBM) antibody disease is an extremely rare disorder. It may be idiopathic or secondary to exposure of the proximal tubular basement membrane, triggered by tubular injury due to acute pyelonephritis, acute allergic interstitial nephritis, or kidney allograft rejection. The histopathology of anti-TBM antibody disease is characterized by strong linear deposits of IgG with complement C3 along the proximal tubular cell basement membranes. The staining is restricted to proximal tubules. Currently, a kidney biopsy with these pathognomonic findings is the only diagnostic method. Serological testing and titers for anti-TBM antibodies are not clinically standardized. Our patient had pyelonephritis and possibly acute allergic interstitial nephritis as a result of nivolumab infusion. The kidney biopsy demonstrated dense interstitial infiltrates of neutrophil-rich interstitial inflammation, neutrophilic casts, and neutrophilic tubulitis consistent with acute pyelonephritis, as well as areas of mixed inflammation with lymphocytic tubulitis suggesting concurrent acute interstitial nephritis. The presence of linear IgG staining along proximal but not distal tubular basement membranes was diagnostic of anti-TBM antibody disease, favored to be due to both triggers. The patient was treated with discontinuation of nivolumab, intravenous antibiotics, and corticosteroids and was supported with hemodialysis. After 6 weeks, the patient's kidney function recovered enough to discontinue hemodialysis and had significant renal improvement.

## 1. Introduction

Acute kidney injury, AKI, as a result of anti-TBM antibody disease is an extremely rare disorder [1]. The basement membrane of the renal tubular cells is rarely exposed. Although immunogenic, pathological conditions related to formation of antitubular basement membrane antibodies are very rare with descriptions limited to case reports [2, 3]. Anti-TBM antibody disease results from exposure of tubular proteins due to proximal tubular damage. It is extremely rare as a “primary” disease and is caused by an autoantibody against a protein called the tubulointerstitial nephritis antigen. This antigen is unique to the kidney and the renal tubules; hence, it is renal-limited. It is a 58 kDa noncollagenous protein expressed within the proximal convoluted tubules.

While it is unusual for anti-TBM disease to be “primary,” multiple “secondary” causes of anti-TBM antibody disease have been described. Secondary causes include lupus nephritis [4], membranous nephropathy, acute rejection of kidney allograft [5], acute interstitial nephritis, or pyelonephritis. Acute rejection with disruption of the integrity of the tubules or absence of the specific antigen in the transplanted kidney [5] has also been reported to stimulate production of anti-TBM antibodies in transplant recipients [3, 6, 7]. Additionally, there are reported cases where antiglomerular basement membrane (GBM) antibodies bind to the tubular basement membrane. These antibodies have been reported in 50–70% of patients with anti-GBM disease [3, 8, 9]. There are also reports of cross reactivity of the anti-GBM antibodies and TBM antigens [9].

The histopathology of anti-TBM antibody disease is characterized by strong linear deposits of IgG with complement C3 along the proximal tubular cell basement membranes [3], with an associated mixed cellular infiltrate of T lymphocytes, plasma cells, and macrophages. Linear IgG staining is restricted to proximal tubules, with no distal tubular epithelial staining or staining within glomeruli.

Our case is a patient who presented with AKI, who has been exposed to nivolumab for the treatment of urothelial malignancy. The patient's presentation was very subtle with a rising serum creatinine and no active urinary sediment. The patient's percutaneous kidney biopsy demonstrated acute neutrophil-rich interstitial inflammation with neutrophilic and lymphocytic tubulitis, and linear IgG and C3 staining by immunofluorescence, diagnostic for anti-TBM antibody disease in the setting of acute pyelonephritis and possible nivolumab nephrotoxicity.

## 2. Case Presentation

This patient was a 71-year-old male who was receiving nivolumab infusions for treatment of urothelial carcinoma who presents with acute kidney injury. The patient's initial cancer diagnosis was 20 months' prior, and bladder biopsy had revealed high grade carcinoma with lymphovascular invasion. The patient was initially treated with gemcitabine and cisplatin for 4 cycles, followed by radical cystectomy with an ileal conduit. The patient was then initiated on nivolumab infusions which was administered every 28 days. The patient had been responding well with near resolution of hypermetabolic abnormalities on positron emission tomography (PET) scans.

The patient was due for his 16th cycle but experienced night sweats, fatigue, and decreased appetite for 2-3 weeks. The patient's infusion was postponed and was found to have acute kidney injury. Laboratory studies showed a blood urea nitrogen (BUN) of 68 mg/dL, serum creatinine 4.4 mg/dL (from a baseline of 1.2 mg/dL with a baseline glomerular filtration rate (GFR) of 60 mL/min), in addition to new-onset leukocytosis with a WBC of 11.8 K/ul. The patient was sent to the emergency department the following day after repeat labs confirmed these abnormalities.

The patient's evaluation in the emergency department revealed a further decline in his renal function with a serum creatinine now up to 5.4 mg/dL; continued mild leukocytosis and computerized tomography of the abdomen and pelvis without intravenous or oral contrast (CT) revealed perinephric stranding without hydronephrosis or apparent ileostomy malfunction. A urine sample was obtained from the patient's urostomy bag revealing 554 WBCs/hpf, 16 RBCs/hpf, many bacteria, rare WBC clump, and 1+ protein. Urine eosinophil stain was negative. The patient was hemodynamically stable, afebrile, and not particularly ill-appearing. The patient was admitted to the hospital and intravenous normal saline for hydration were initiated and the patient received intravenous Ceftriaxone after blood cultures and urine cultures were obtained.

The patient's home medications were benazepril 10 mg daily, apixaban 5 mg twice a day for atrial fibrillation, and simvastatin 20 mg daily. Urine culture was positive for pan sensitive *E. coli*. Blood cultures were negative. Despite hydration and intravenous antibiotics, the patient's renal function continued to decline. The patient developed volume overload with small bilateral pleural effusions and hyponatremia. The patient continued to make a fair amount of clear yellow urine throughout his stay but developed oliguria on hospital day 4. Dialysis was initiated on hospital day 5 at which point the patient's BUN and creatinine had risen to 82 mg/dL and 9.6 mg/dL, respectively.

**Figure 1:**
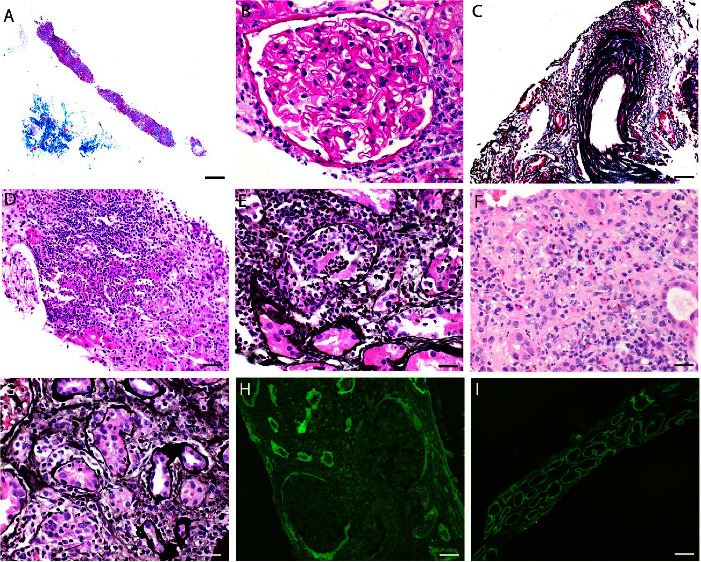
Histopathologic findings on kidney biopsy. (a) Moderate interstitial fibrosis and tubular atrophy throughout cortex (masson trichrome stain, 20x magnification, scale bar = 500 *μ*m). (b) Unremarkable glomerulus (periodic acid Schiff stain, 400x magnification, scale bar = 20 *μ*m). (c) Interlobular artery with severe intimal fibrosis (SMMT stain, 400x magnification, scale bar = 20 *μ*m). (d) Neutrophil-rich infiltrate within tubulointerstitium (hematoxylin and eosin stain, 200x magnification, scale bar = 50 *μ*m). (e) Neutrophilic tubulitis (jones methenamine silver stain, 600x magnification, scale bar = 20 *μ*m). (f) Mixed interstitial inflammation with lymphocytes, plasma cells, eosinophils, histiocytes, and focal neutrophils (hematoxylin and eosin stain, 600x magnification, scale bar = 20 *μ*m). (g) Lymphocytic tubulitis (jones methenamine silver stain, 400x magnification, scale bar = 20 *μ*m). (h) Linear IgG staining along proximal tubular basement membranes (fluorescein-conjugated antihuman IgG stain, 100x magnification, scale bar = 100 *μ*m), with lack of staining along glomerular basement membranes. (i) Linear IgG staining along proximal tubular basement membranes (fluorescein-conjugated antihuman IgG stain, 100x magnification, scale bar = 100 *μ*m).

**Figure 2:**
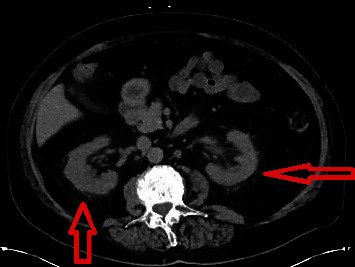
Computerized tomography of the abdomen and pelvis without contrast demonstrating bilateral perinephric stranding (red arrows), consistent with acute pyelonephritis.

A kidney biopsy was performed to identify the etiology of the patient's acute kidney injury. The biopsy had 17 glomeruli, of which 3 were globally sclerotic. The glomeruli had an unremarkable appearance, without mesangial expansion, mesangial hypercellularity, endocapillary hypercellularity, fibrinoid necrosis, karyorrhexis, or crescent formation. There was moderate interstitial fibrosis and tubular atrophy with a dense interstitial infiltrate of mixed interstitial inflammation, comprised of eosinophils, neutrophils, lymphocytes, plasma cells, and histiocytes within both intact and atrophic kidney parenchyma. There was patchy, but dense neutrophil-rich interstitial inflammation accompanied by neutrophilic rimming along the tubules and white blood cell casts (Figure 1). Both neutrophilic and lymphocytic tubulitis was identified. Immunofluorescence microscopy demonstrated strong linear IgG and C3 staining (3+ each) along the proximal tubular basement membranes without associated glomerular or distal tubular epithelial staining (Figure 2). IgG subclass staining revealed staining for all IgG subclasses along the TBMs (3+ each for IgG1, IgG2, IgG3, and IgG4). Electron microscopy did not reveal tubular basement membrane deposits.

This patient had a neutral urine except for urinary tract infection, especially when cultures grew *E. coli*. The CT scan of the abdomen and pelvis without contrast was suggestive of pyelonephritis, as it had stranding in the perinephric space (Figure 2). Serological studies for antinuclear antibodies, ANA, and antineutrophilic cytoplasmic antibodies, ANCA, were negative. Antiglomerular basement membrane antibodies were negative. Serologic testing for anti-TBM antibodies is not a routine clinical laboratory test, and therefore, was not tested initially. Even if this test was available and was positive, it is unclear whether there is an association between antibody titers and disease activity, as there is insufficient data available and no validated clinical assays.

The patient was initiated on intravenous methylprednisolone 40 mg every 8 hr but continued to require hemodialysis. A permcath was placed and the patient was eventually discharged home on prednisone 60 mg orally, daily, and continued intravenous Ceftriaxone 2 gram daily for an additional 2 weeks and scheduled for 3 times weekly hemodialysis.

After discontinuation of nivolumab, intravenous antibiotic therapy, and continued steroid therapy (prednisone 60 mg/day), and during that time, the patient was being supported by hemodialysis, the patient's kidney function gradually improved and prednisone dose was slowly tapered. The patient's renal function improved and hemodialysis was no longer required.

Serologic testing for anti-TBM antibodies by indirect immunofluorescence was performed and was negative, suggestive of serologic remission. The patient was 6 weeks of being on steroids, nivolumab discontinued, and intravenous antibiotics. Baseline titers were not available for comparison.

## 3. Discussion

The CT scan findings, positive urine cultures, and histopathologic changes on kidney biopsy all support the diagnosis of acute pyelonephritis. However, AKI may have been temporally correlated with drug exposure to the patient's chemotherapy. It is possible, and likely, that both pyelonephritis and nivolumab exposure inducing acute allergic interstitial nephritis led to tubulointerstitial injury that resulted in TBM exposure and development of anti-TBM antibodies. There was evidence to support concurrent nivolumab nephrotoxicity due to kidney biopsy findings of mixed interstitial inflammatory infiltrates including eosinophils and lymphocytic tubulitis.

Nephrotoxicity is a well-recognized complication of use of checkpoint inhibitor therapy (including nivolumab), which most commonly manifests with acute or chronic tubulointerstitial nephritis [10]. The overall frequency of checkpoint inhibitor nephrotoxicity has been reported as 3–5%, which is likely underestimated as less than 10% of patients with AKI due to checkpoint inhibitor therapy undergo kidney biopsy [11].

Risk factors for nephrotoxicity include pre-existing renal impairment, older age, and concomitant use of other nephrotoxic agents. Histopathological features of nivolumab-induced nephrotoxicity may vary depending on the presentation of the toxicity. Acute interstitial nephritis is the most common presentation [12] and is characterized by interstitial edema, infiltration of inflammatory cells (such as eosinophils), and tubular injury [13]. In glomerulonephritis, there may be varying degrees of glomerular injury, such as segmental or global sclerosis, crescent formation, or mesangial proliferation. Nephrotic syndrome may be due to minimal change disease or focal segmental glomerulosclerosis. Acute kidney injury may show acute tubular necrosis or acute interstitial nephritis [11]. Vasculitis and other glomerular diseases, including podocytopathies and C3 glomerulonephritis, are other rare adverse events that were not observed in our patient [12].

The pathogenesis of anti-TBM antibody disease is not directly related to nivolumab-induced nephrotoxicity. However, the immune-mediated mechanisms of nivolumab-induced nephrotoxicity, such as T-cell activation and cytokine release, can lead to inflammation and damage to renal tissue, and may have some overlap with the pathogenesis of anti-TBM antibody disease by exposing the basement membrane of the proximal tubules. Further research is needed to elucidate the precise mechanisms underlying both conditions [11].

We chose not to perform therapeutic plasma exchange (TPE). While, theoretically, anti-TBM antibody disease may respond to TPE, there is no clear evidence in the literature of its efficacy. Additionally, there were “secondary” triggers for the patient's disease, in which mitigating these factors would be important to control ongoing tubulointerstitial injury.

## 4. Conclusion

AKI as a result of anti-TBM antibody disease is an extremely rare disorder. The diagnosis requires kidney biopsy. Anti-TBM antibody titers are not routinely measured in clinical practice and the presence of titers may not correlate with the disease activity. Given the rarity of the disease, anti-TBM serology would not serve as a useful screening test.

Acute allergic interstitial nephritis, pyelonephritis, and kidney allograft rejection can act as “secondary” triggers for the disease and this case is the second reported case of pyelonephritis associated with development of anti-TBM antibody disease, with no prior reports of checkpoint inhibitor therapy as a contributing factor.

As anti-TBM antibody disease is exceedingly rare, optimal treatment modalities are uncertain. There is anecdotal evidence for corticosteroid therapy in addition to abating “secondary” triggers of disease. Further data is required to determine the best course of treatment, such as whether removal of anti-TBM antibodies through TPE has any clinical benefit. We encourage others to share their experience by reporting their patients or ideally through multi-institutional collaboration to improve our understanding of this rare disease.

## Data Availability

No data were analyzed or generated during this study.
